# Quantifying discrepancies between EMS time intervals in the Swedish Registry for Cardiopulmonary Resuscitation and emergency medical dispatch data: a 10-year analysis

**DOI:** 10.1016/j.resplu.2026.101534

**Published:** 2026-10-20

**Authors:** Erik Boberg, Carl Magnusson, Douglas Spangler, Fredrik Byrsell, Martin Jonsson

**Affiliations:** a Department of Clinical Science and Education, Södersjukhuset, Centre for Resuscitation Science, Karolinska Institute, Stockholm, Sweden; b Department of Prehospital Emergency Care, Sahlgrenska University Hospital, Gothenburg, Sweden; c Department of Molecular and Clinical Medicine, Institute of Medicine, Sahlgrenska Academy, University of Gothenburg, Gothenburg, Sweden; d PreHospen-Centre for Prehospital Research, Faculty of Caring Science, Work Life and Social Welfare, University of Borås, Borås, Sweden; e Uppsala Centre for Prehospital Research, Department of Surgical Sciences – Anesthesiology and Intensive Care, Uppsala University, Uppsala, Sweden

**Keywords:** Out-of-hospital cardiac arrest, Emergency medical services, Swedish Registry for Cardiopulmonary Resuscitation, Response time, Cardiac arrest registry, Dispatch centre

## Abstract

**Objective:**

To assess discrepancies between time intervals reported to the Swedish Registry for Cardiopulmonary Resuscitation (SRCR) for out-of-hospital cardiac arrest (OHCA) and those recorded by the dispatch centre between 2015 and 2024.

**Methods:**

In this retrospective observational study, OHCAs reported to the registry were linked to dispatch centre data to correct and validate EMS case numbers. Time interval discrepancies were quantified by comparing the EMS dispatch-to-arrival interval (unit response time) with the call start-to-arrival interval (total response time). Differences between the two sources were estimated using Bayesian quantile regression by modelling the median response-time distribution.

**Results:**

Among 56,969 registry records, 1004 (1.8%) lacked an EMS case number. On a pooled national level, unit response time showed negligible discrepancies between the two sources, with a median difference of 0.3 s (95% CrI −4.1 to 3.9). In contrast, the total response time was systematically shorter in the registry, with a median difference of −80.9 s (95% CrI −84.7 to −77.1). Both time intervals showed large regional variation, with the smallest difference in the Stockholm region.

**Conclusion:**

On a pooled national level, unit response time showed high concordance, whereas total response time was systematically shorter in the registry. Registry time variables, particularly total response time, should be interpreted with caution when evaluating small performance differences.

## Introduction

Out-of-hospital cardiac arrest (OHCA) remains a leading cause of death in Europe.^1^ Survival is time-dependent,^2^ relying on early recognition, bystander cardiopulmonary resuscitation (CPR), defibrillation, and rapid Emergency Medical Services (EMS) response.^3^, ^4^ The European Resuscitation Council (ERC) highlights cardiac arrest registries as a cornerstone of quality improvement and system evaluation.^5^

Sweden is one of a few European countries with a nationwide OHCA registry covering the entire population.^6^ The Swedish Registry for Cardiopulmonary Resuscitation (SRCR), established in 1990, is a national registry for all OHCAs in Sweden.^7^ It enables surveillance and benchmarking of outcomes across 21 healthcare regions with multiple EMS providers.

EMS response time is a key performance indicator in OHCA. In the 2024 Utstein template,^8^ the recommended standard interval is the time from when the call is connected to the primary public safety answering point (PSAP) until the arrival of the first EMS unit at the scene (total response time). An alternative measure is the interval from EMS dispatch to arrival at the scene, hereafter referred to as the unit response time.

Over recent decades, EMS response times in Sweden have nearly doubled.^9^ Accurate measurement of these intervals is therefore important for monitoring system performance and for interpreting temporal and regional differences. While some cardiac arrest registries use automated, system-derived timestamps from the dispatch centre, the Swedish registry, relies on manually entry by the EMS crew. To our knowledge, these manually reported timestamps have not been validated against an external data source. Consequently, the data may be vulnerable to recall bias, rounding errors, and potential misrepresentation of the true operational performance.

To address this knowledge gap, this study primarily aimed to assess discrepancies between manually recorded registry times and digitally recorded timestamps from dispatch centres across Sweden by year, region, and time of day. A secondary aim was to examine the completeness and correctness of EMS case numbers reported in the registry.

## Methods

### Study design and setting

This retrospective observational study evaluated discrepancies between time intervals reported to the SRCR and time intervals recorded by the dispatch centre. The study was conducted in Sweden, with a population of 10.6 million in 2025,^10^ between January 1, 2015, to December 31, 2024.

Ethical approval was granted by the Swedish Ethical Review Authority (dnr 2025-03753-01). Owing to the retrospective design and use of previously collected data, the requirement for informed consent was waived.

### Data sources

#### The Emergency Medical Service organisation

Sweden has a regionally organised EMS system, distributed across 21 healthcare regions. EMS units are typically staffed by a registered nurse and an emergency medical technician.

#### The Emergency medical dispatch centres

Sweden uses a centralised public safety answering point (PSAP) system managed by SOS Alarm AB, which manages all 112 emergency calls nationwide. In most regions (17/21), they are also responsible for dispatch operations, creating a one-step dispatch system. In four regions, the dispatch operations are organised independently under the name of Sjukvårdens larmcentral (SvLc), creating a two-step dispatch organisation. In these regions, the dispatch centres are integrated within the EMS organisation. If a patient is unconscious with abnormal breathing, all dispatch centres report the incident as a suspected cardiac arrest.

#### The Swedish Registry for Cardiopulmonary Resuscitation

The SRCR was created in the early 1990s. Since 2010, all EMS organisations report EMS-treated OHCAs to the registry. The registry is hosted by the Centre of Registries, Västra Götaland, which provides technical and legal support for infrastructure and governance. Patient data is reported using a standardised resuscitation template^11^ based on the Utstein recommendations.^8^ Through annual reports, the registry describes temporal trends in resuscitation care and provides comparisons across all healthcare regions. Survival at 30-days is obtained automatically through linkage with the Swedish Population Registry.

#### Data generation and registry entry

All dispatch centre timestamps are generated in real time within a computer-aided dispatch system, that logs the call start time (PSAP) and the EMS dispatch time. The EMS crew receives mission details and the dispatch centre timestamps via their in-vehicle terminals. There is no automated linkage between EMS operational databases and the registry. Apart from the Region of Stockholm, registry entry is performed manually via a web portal after mission completion, sometimes several days later, and are largely based on manual retrospective documentation. Finally, in multi-unit responses, the dispatch system records the arrival time of the first responding vehicle. In contrast, the registry report may be completed by a secondary responding unit, reporting its own arrival time.

#### Study population

All registry records between January 1, 2015, and December 31, 2024, were included. No exclusion criteria were applied.

#### Study objective

The primary objective was to assess the potential discrepancies between time intervals reported to the registry and those recorded by the dispatch centre. The time intervals of interest were the total response time (PSAP to EMS arrival) and the unit response time (EMS dispatch to EMS arrival). To accurately link the registry records with the dispatch centre data, a correct EMS case number had to be verified. Therefore, the secondary aim was to examine the proportion of correct EMS case numbers in the registry.

#### Record linkage

Registry records were linked to dispatch centre data using a strict hierarchical matching process. First, records with identical case numbers were fully matched. Second, partial case-number matching was used to account for common formatting variations (missing prefixes or suffixes). Third, for records with incomplete or non-matchable case numbers, manual review was conducted to identify the most plausible dispatch centre record. A case was considered a match when a OHCA in the registry and a suspected cardiac arrest at the dispatch centre occurred on the same day and in the same region/municipality within an narrow time window (e.g., registry time 02:01 and a dispatch centre call logged at 02:00:00). Cases matched through manual review, along with the partial and fully matched categories, were included in the final denominator. To minimise misclassification, cases with unclear identifiers that could not be resolved through manual review were excluded, ensuring that the analysed cohort comprised only correct matches. (Technical details on case-number format and dispatch centre variations are provided in Supplementary Table S1).

#### Calculation of time intervals in SRCR and the dispatch centre

After linking the registry case numbers with the dispatch records, response time intervals were calculated separately for each data source. For both the registry and dispatch centre data, the unit response interval was calculated as the time from EMS dispatch to EMS arrival at the scene. The total response time interval was calculated as the time from the PSAP to EMS arrival at the scene. All intervals were calculated in seconds, and the difference between the two sources was compared as (registry – dispatch centre).

### Statistical analysis

The proportion of correctly registered OHCA cases in the registry is reported as the percentage of agreement between the originally recorded EMS case number and corrected case numbers. Agreement is reported for both fully and partially corrected case numbers. A Bayesian framework was chosen to provide an intuitive probabilistic interpretation of the interval estimates. To quantify discrepancies in unit and total response time between the registry and the dispatch centre, we modelled these differences using Bayesian quantile regression with an asymmetric Laplace likelihood. For each time interval, we fitted intercept-only models to estimate the population-level median for both sources. We used default priors (non-informative) for the asymmetric Laplace distribution. The median difference was derived by subtracting posterior draws from the dispatch centre model from those of the registry model (registry – dispatch centre), yielding a posterior distribution for the median difference. All analyses were repeated by calendar year, healthcare region, time of day and day of the week. Results are reported as the posterior median difference with 95% credible intervals (CrI), together with the full posterior distribution. Additionally, we calculated the posterior probability that the differences exceeded a clinically relevant threshold of 60 s.

For each model, we ran ten parallel Markov chain Monte Carlo (MCMC) chains, each consisting of 4000 iterations with a warm-up period of 1000, resulting in a total of 30,000 post-warm-up draws for each analysis. Model convergence was assessed using the Gelman-Rubin diagnostic (R-hat), ensuring all values were <1.01, and by verifying that the Effective Sample Sizes (ESS) were >1000 for all parameters. Statistical analysis was performed using R version 4.4.2 (R Foundation for Statistical Computing, Vienna, Austria). The Bayesian regression analyses were performed using the brms package.^12^ Analytic data, together with the code required to reproduce the analyses and figures, are available on the Open Science Framework (https://doi.org/10.17605/OSF.IO/DQJA5).

## Results

Between 2015 and 2024, 57,708 OHCAs were reported to the registry. After removal of duplicate entries, 56,969 remained for analysis. Among these, 1004 (1.8%) had no recorded EMS case number.

### Pooled response interval discrepancies

For the unit response interval, the median difference between the registry and the dispatch centre was 0.3 s (95% CrI: −4.1 to 3.9), with 0% probability of exceeding a 60-s difference. For the total response interval, the registry time was shorter than that of the dispatch centre, with a median difference of −80.9 s (95% CrI: −84.7 to −77.1) and a 100% probability that the difference exceeded 60 s (Fig. 1).

**Fig. 1:**
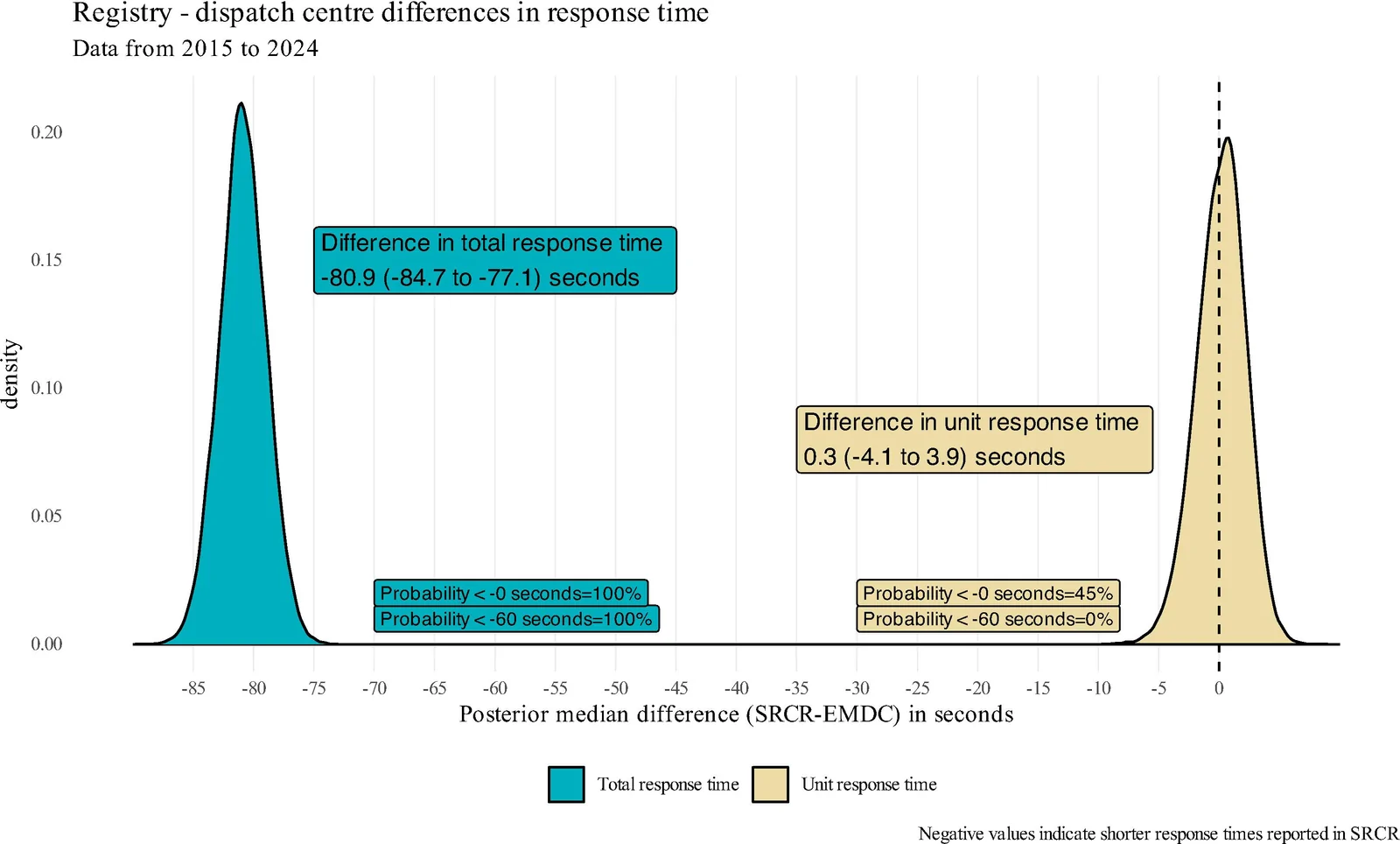
**Systematic discrepancy in unit and total response time between the registry and dispatch centre (2015–2024)**. *Pooled posterior distributions of the median difference in response times between registry and dispatch centre for 2015–2024. The x-axis represents the difference in seconds (registry – dispatch centre). Negative values indicate that the response times reported in the registry are shorter than those in the dispatch centre. The total response interval shows a clear negative shift with a median difference of −80.9 s and a 100% probability of exceeding a 60 s difference. In contrast, the unit response interval is centred near zero, indicating a negligible systematic offset with a 0% probability of exceeding the 60-s threshold*. *SRCR = Swedish Registry for Cardiopulmonary Resuscitation, EMDC = Emergency medical dispatch centre*.

### Regional variations in discrepancies

Regional analyses showed that the differences in unit response interval varied across healthcare regions. The smallest difference was observed in Stockholm (−4 s, 95% CrI: −13 to 8), followed by Central Sweden (−20 s, 95% CrI: −29 to −11) and Western Sweden (−51 s, 95% CrI: −58 to −43). The largest discrepancy was observed for Southern Sweden (−74 s, 95% CrI: −83 to −62).

For the total response interval, discrepancies were larger. Stockholm showed the least evidence of a systematic difference (−32 s, 95% CrI: −39 to −26), whereas all other regions had median differences exceeding −70 s, with the largest difference observed in Central Sweden (−132 s, 95% CrI: −143 to −121) (Fig. 2).

**Fig. 2:**
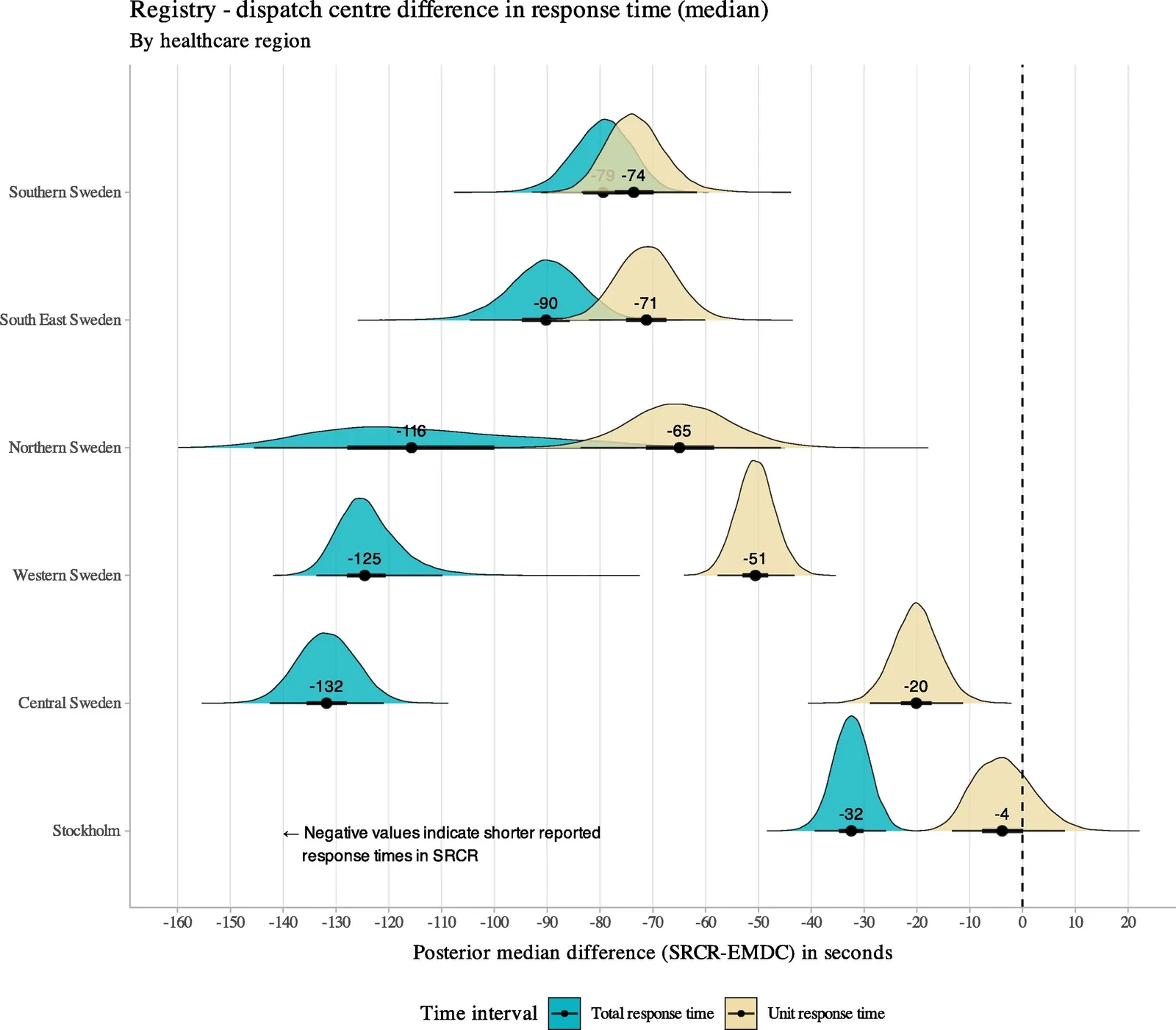
**Discrepancies in total and unit response time by healthcare region**. *Posterior distributions of the median difference in response times between the registry and the dispatch centre, by healthcare region. The x-axis shows registry – dispatch centre in seconds; negative values indicate shorter response times reported in the registry compared to the dispatch centre. Teal distributions represent the total response time, and beige distributions represent the unit response time. Points and horizontal lines indicate the posterior median and 95% credible interval for each region. Registry total response times are systematically shorter than the dispatch centre across all regions, whereas unit response times show smaller and more variable differences*. *SRCR = Swedish Registry for Cardiopulmonary Resuscitation, EMDC = Emergency medical dispatch centre*.

### Annual trends in discrepancies

Year-specific estimates are shown in Fig. 3. The median unit response interval difference ranged from −44 s in 2019 (95% CrI: −63 to −4) to approximately zero in 2020. For the total response interval, the largest discrepancy occurred in 2015, when response times in the registry were more than 2 min shorter than those in the dispatch centre (−133 s, 95% CrI: −147 to −119). The smallest difference occurred in 2024 (−67 s, 95% CrI: −81 to −54).

**Fig. 3:**
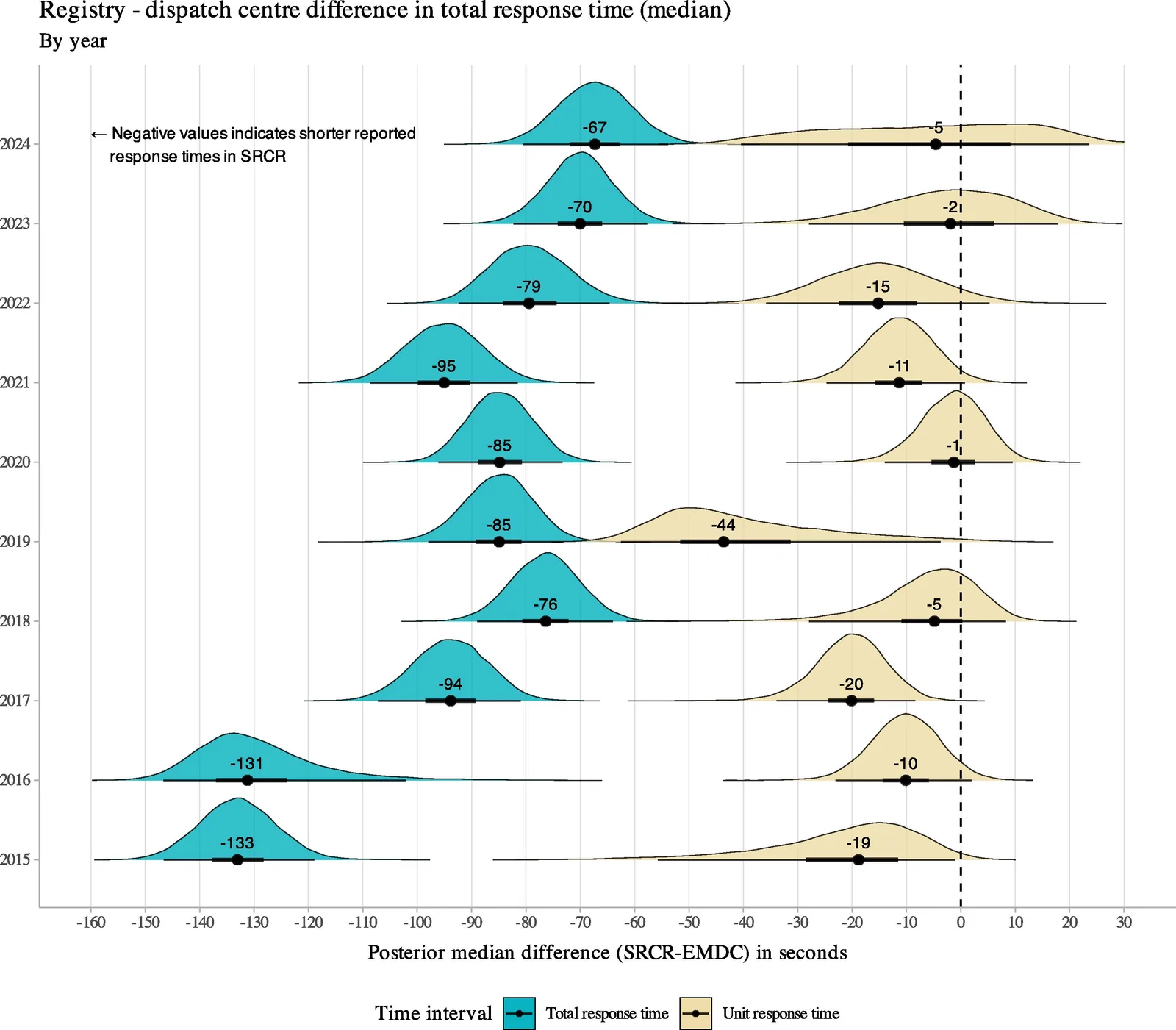
**Yearly discrepancy in total and unit response time between the registry and dispatch centre (2015–2024)**. *Posterior distributions of the yearly median difference in response times between the registry and the dispatch centre, 2015–2024. The x-axis shows registry – dispatch centre in seconds; negative values indicate shorter response times reported in the registry than in the dispatch centre. Teal represents the total response time, and beige represents the unit response time. For each year, the point and horizontal line show the posterior median and 95% credible interval. Across all years, total response time is systematically shorter in the registry than in the dispatch centre, whereas unit response time shows smaller and often near-zero discrepancies*. *SRCR = Swedish Registry for Cardiopulmonary Resuscitation, EMDC = Emergency medical dispatch centre*.

### Discrepancies in response intervals by time of day

In Fig. 4, the time intervals are presented by time of day. For the unit response interval, the largest discrepancies were observed between 15:00–17:59 with a median difference of −45 s (95% CrI: −54 to −33). The smallest discrepancy was observed between 06:00 and 08:59 with a median difference of −7 s (95% CrI: −17 to 1). Variations were larger for the total response interval*,* ranging from −107 s (during both 21:00–23:59 and 03:00–05:59) to −73 s (during 15:00–17:59).

**Fig. 4:**
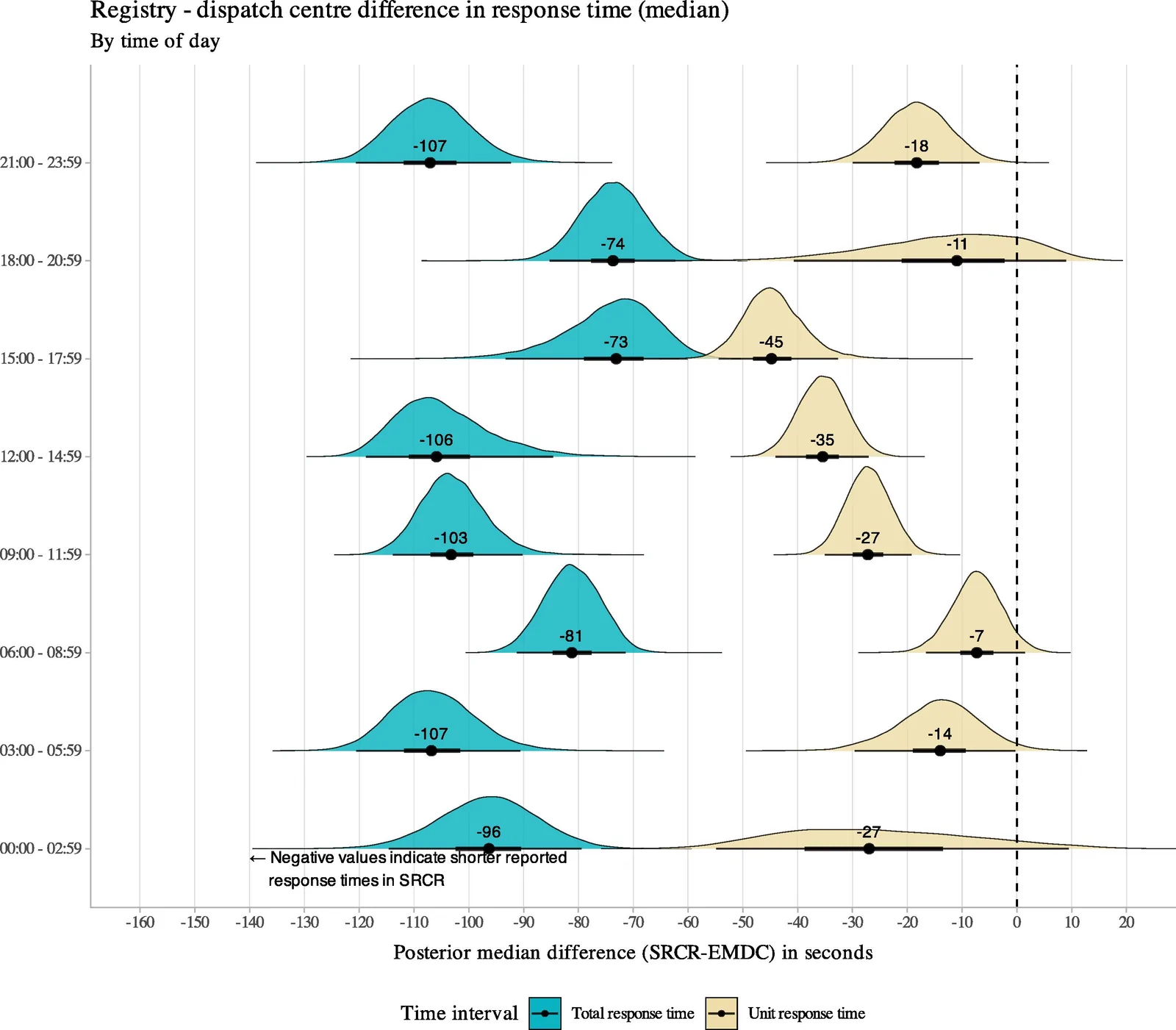
**Discrepancies in response intervals by time of day (2015–2024)**. *Posterior distributions of the median response time differences (registry – dispatch centre) in seconds, categorised by 3-hour time intervals. Negative values indicate that the registry recorded shorter response times than the dispatch centre. Teal represents the total response time, and beige represents the unit response time. Points and horizontal lines indicate the posterior median and 95% credible interval*. *SRCR = Swedish Registry for Cardiopulmonary Resuscitation, EMDC = Emergency medical dispatch centre*.

### Discrepancies in response intervals by day of the week

The time intervals by day of the week are presented in Fig. 5. For the unit response interval*,* discrepancies remained small across all days of the week except Saturday, with a median difference of −47 s (95% CrI: −57 to −36). The discrepancy for the total response interval was larger, ranging from −92 s on Sundays to −75 s on Fridays.

**Fig. 5:**
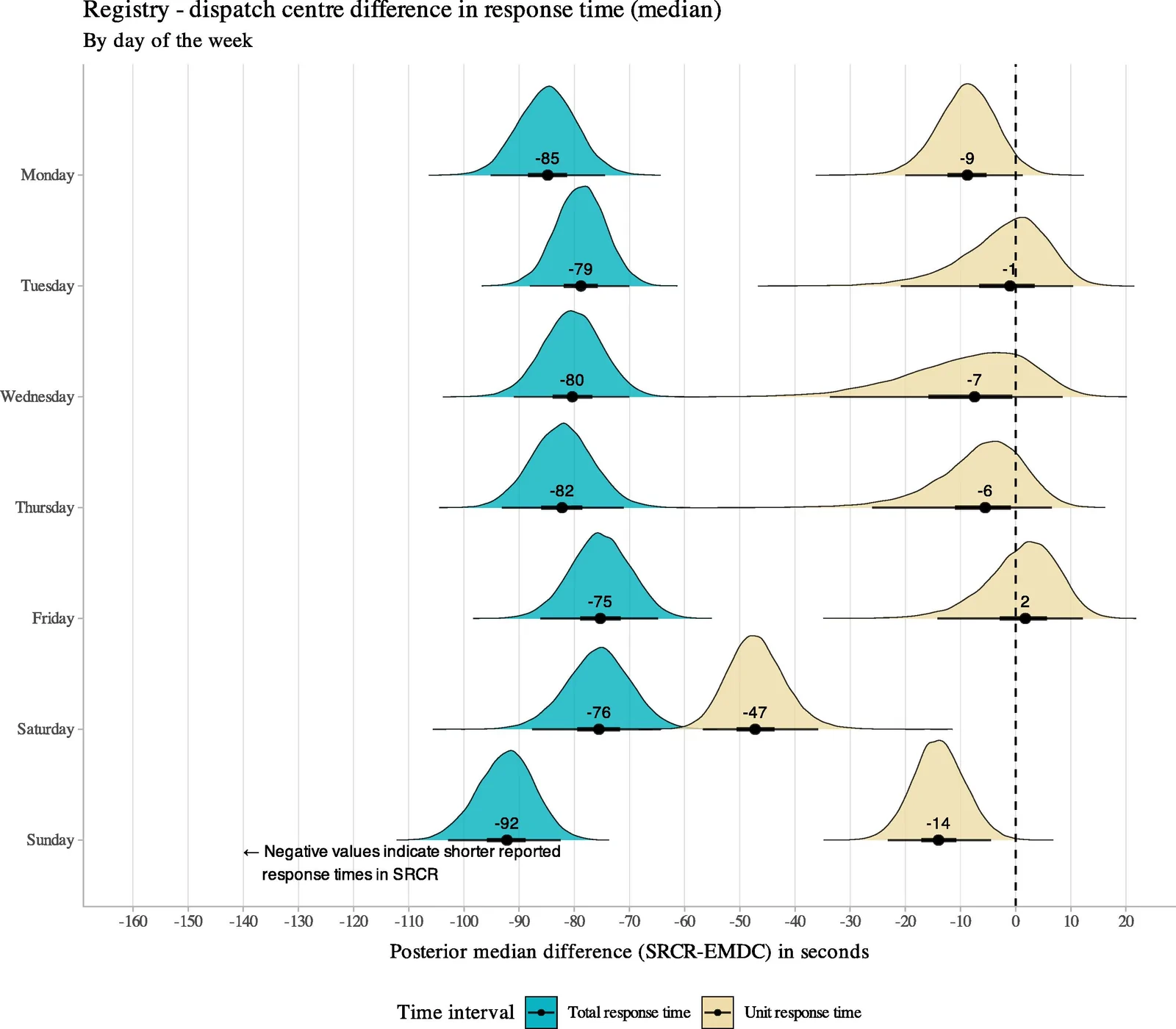
**Discrepancies in response intervals by day of the week**. *Posterior distributions of the median response time differences (registry – dispatch centre) in seconds, divided by day of the week. Negative values indicate that the registry recorded shorter response times than the dispatch centre. Teal represents the total response time, and beige represents the unit response time. Points and horizontal lines indicate the posterior median and 95% credible interval*. *SRCR = Swedish Registry for Cardiopulmonary Resuscitation, EMDC = Emergency medical dispatch centre*.

### Valid EMS case number

The proportion of fully correct EMS case numbers varied by healthcare region. Two regions had 0% fully correct case numbers, whereas Stockholm had the highest proportion of fully correct case numbers at 98%. For partially correct case numbers (missing dispatch centre code), the proportion also varied by region, ranging from 77% in Central Sweden to 100% in Stockholm (Supplementary Fig. S1). Across the entire cohort, the proportion of registry records with partially correct case numbers was approximately 90% until 2020 but declined in later years to around 85% in 2022–2024 (Supplementary Fig. S2).

## Discussion

The present study investigated discrepancies between the EMS time intervals reported to the Swedish Registry for Cardiopulmonary Resuscitation and those recorded at the dispatch centre. The total response time interval is considered the gold standard according to the Utstein guidelines.^8^ We found a large discrepancy between the total response time reported in the registry and the time recorded at the dispatch centre (with a 100% probability of exceeding the 60-s threshold), suggesting a consistent, systematic offset. The systematic discrepancy is likely driven by variations in how time variables are reported to the registry. While the disptach centre automically logs system-generated timestamps, registry entries rely on retrospective and manually reported timestamps (apart from the region of Stockholm). Additionally, in multi-unit responses the dispatch centre logs the arrival time of the first arriving unit, whereas the registry entry may be completed by a subsequent unit, recording its own arrival time. Conversely, the unit response time demonstrated high concordance at the pooled national level (with a 0% probability of exceeding 60 s) likely beacuse both sources rely on a shared, system generated EMS disaptch timestamp.

The increase in discrepancies during rush hours (15:00–17:59) and weekends supports this explanation. During high-stress, high-volume periods, EMS crews naturally prioritise clinical care over administrative duties. In contrast, in Region Stockholm, where registry reporting is mandatory before the next mission, the smallest discrepancies in both unit and total response time were observed. However, without an independent reference standard, the direction and cause of this discrepancy cannot be determined with certainty in this study.

The unit response time showed high agreement when all data were pooled, but the underlying discrepancies appear when the data are broken down by year and region. This is due to the skewed distribution of the time variables and reflects Simpson’s paradox (a statistical phenomenon where the pooled results can differ from, or even reverse, the patterns observed when the data are stratified by a third variable).

The SRCR remains a valuable source for epidemiological research and quality improvement; however, its time variables should be interpreted with caution, especially in analyses where small differences may affect study conclusions.

Systems that incorporate routine linkage between cardiac arrest registries and computer-aided dispatch systems are likely to provide more robust data and reduce the administrative burden on EMS crews by eliminating the need for retrospective manual timestamp entry. A practical approach to improve future accuracy for non-automated registries would be to integrate GPS-based geofencing, where arrival at the scene is automatically recorded when an EMS vehicle enters a predefined radius around the coordinates of the OHCA. This would provide an objective timestamp that mitigates the risk of approximation, recall bias or rounding errors.

The absence of fully automated data linkage between the dispatch centre and the SRCR reflects structural and resource limitations. Establishing a unified, automated pipeline from the dispatch centres and regional EMS databases to the SRCR requires technical coordination and administrative resources that have not yet been realised.

Our findings are particularly relevant for international registries that rely on manual timestamp recording. To improve registry data quality and address the discrepancies identified in this study, we propose three primary strategies: First, standardised recording of EMS case numbers to facilitate seamless data linkage. Second, direct integration of automated dispatch timestamps, supplemented by GPS-based tracking, to eliminate manual recording of EMS arrival times. Third, a general transition toward automated, direct data transfer from dispatch and EMS databases into the registry.

### Limitations

This work should be interpreted in light of several limitations. First, dispatch centre timestamps are system-generated during call handling and may still contain recording errors. Therefore, our findings should be interpreted as a measure of concordance between two routinely collected data sources rather than a measure of absolute truth. Without an independent reference standard, we cannot definitively establish absolut accuracy; however automated distpach data is less prone to retrospective and manual entry, that affects registry timestamps.

Second, the lack of a common unique identifier, in a standardised format, between the registry and the dispatch centre necessitated a manual linkage process. While we applied a strict, rule-based process to ensure correct matching, the absence of an automated linkage key remains a methodological limitation. Manual linkage may introduce a small risk of incorrect matches, especially in rare circumstances with multiple OHCA events in close temporal proximity within the same region. However, all ambiguous duplicates identified by manual review were excluded to preserve the validity of the final cohort.

Finally, the dispatch centre data do not provide a population denominator for incidence, and we could not estimate national case completeness of the registry, because the number of EMS-treated OHCAs missing from the registry is unknown.

## Conclusion

Unit response time demonstrated high overall agreement with dispatch data, while total response time was systematically underestimated in the registry. Registry-based time variables should be intepreted cautiously when evaluating small performance differences, highlighting the need for automated data integration.

## Disclosures

Carl Magnusson is the registry record keeper for the SRCR. Fredrik Byrsell is a research coordinator at SOS Alarm. Douglas Spangler is responsible for data management and storage at Sjukvårdens Larmcentral. Martin Jonsson is a member of the SRCR Steering Committee.

## CRediT authorship contribution statement

**Erik Boberg:** Writing – original draft, Visualization, Project administration, Methodology, Formal analysis, Data curation, Conceptualization. **Carl Magnusson:** Writing – review & editing, Methodology. **Douglas Spangler:** Writing – review & editing, Methodology. **Fredrik Byrsell:** Writing – review & editing, Methodology. **Martin Jonsson:** Writing – original draft, Visualization, Supervision, Software, Methodology, Formal analysis, Data curation, Conceptualization.

## Declaration of competing interest

All authors declare no conflict of interest.

## Appendix A Supplementary material

Supplementary Data 1 Supplementary material: Contains Table S1 (examples of dispatch centre case number formats and registry variations) and Figures S1-S2 (proportion of correct case number by healthcare region and temporal trends from 2015-2024).
