# Supplementary material for: Quantifying discrepancies between EMS time intervals in the Swedish Registry for Cardiopulmonary Resuscitation and emergency medical dispatch data: a 10-year analysis
Source: Resusc Plus. 2026 Oct 20;32:101534. doi: 10.1016/j.resplu.2026.101534 (PMC13769094; doi:10.1016/j.resplu.2026.101534)
Supplement: Supplementary file 1 [file mmc1.docx]

**Supplement appendix**

**Supplement Table S1:** Examples of corrected dispatch centre case number formats and common registry variants.

| Dispatch centre | *Correct case number* | *Recorded in SRCR* | *Partial match ^*^* | *Fully match* ^†^ |
| --- | --- | --- | --- | --- |
| **SOS alarm** | 19-9876543 | 19-9876543 | √ | √ |
|  |  | 19-98765432-002 | √ | √ |
|  |  | 1998765432 | √ | √ |
|  |  | 98765432-2 | √ | Χ |
|  |  | 98765432 | √ | X |
|  |  | 97654322 | X | X |
|  |  |  |  |  |
| **SvLc** | 20260101-100-26 | 20260101-100-26 | √ | √ |
|  |  | 100-20260101-26-1 | √ | √ |
|  |  | 20260101-100-26-1 | √ | √ |
|  |  | 10020260101261 | √ | √ |
|  |  | 20260101-26 | X | X |
|  |  | 20260101-100 | X | X |

*^*^ Sufficient information to reconstruct and match a unique dispatch centre case number.*

*† Sufficient information to identify a match.*

*Supplement Table S1: The case-number structure used in dispatch centre systems (SOS Alarm and SvLc) and common variants observed in the registry illustrate how EMS crews recorded case numbers in the registry. The table also shows if the registry variant supported a partial or complete (fully) match for case ascertainment. SRCR = Swedish Registry of Cardiopulmonary Resuscitation, SvLc = Sjukvårdens larmcentral.*

**Supplement Figure S1:** Proportion of registry records with fully (complete) and partially correct EMS case numbers, by healthcare region.

*Supplement Figure S1. Proportion of registry records with (A) fully correct and (B) partially correct EMS case numbers, by healthcare region*. *A fully correct EMS case number indicates a complete case number suitable for direct linkage to* dispatch centre *records. A partially correct case number indicates that the main case-number component is present, but the dispatch central code is missing, requiring correction before linkage.*

**Supplement Figure S2.** Temporal trends in fully and partially correct EMS case numbers recorded in the registry, 2015–2024.

*Supplement Figure S2. Proportion of registry records with fully and partially correct EMS case numbers by year (2015–2024). The figure shows consistently high levels of partially correct case numbers through 2020, followed by a modest decline in later years, alongside an increase in fully correct case numbers after 2020. (A fully correct EMS case number indicates a complete case number suitable for direct linkage to* dispatch centre *records. A partially correct case number indicates that the main case-number component is present, but the dispatch central code is missing, requiring correction before linkage).*
